# Spontaneous bladder rupture diagnosis based on urinary appearance of mesothelial cells: a case report

**DOI:** 10.1186/1752-1947-8-46

**Published:** 2014-02-12

**Authors:** Waka Hayashi, Tomoya Nishino, Satoru Namie, Yoko Obata, Masataka Furukawa, Shigeru Kohno

**Affiliations:** 1 Department of Internal Medicine, Sasebo Chuo Hospital, Nagasaki, Japan; 2 Second Department of Internal Medicine, Nagasaki University School of Medicine, Nagasaki, Japan; 3 Medical Education Development Center, Nagasaki University Hospital, Nagasaki, Japan; 4 Department of Urology, Sasebo City General Hospital, Nagasaki, Japan

**Keywords:** Acute renal failure, Mesothelial cells, Spontaneous bladder rupture, Urinary ascites

## Abstract

**Introduction:**

Spontaneous bladder rupture is an extremely rare clinical event that is associated with urinary ascites and apparent acute renal failure. This event is difficult to diagnose clinically, even with advanced techniques such as computed tomography; however, the timely diagnosis of this condition is critical. Here, we report a case of a patient who experienced a spontaneous intraperitoneal bladder rupture 10 years after postoperative pelvic irradiation for the treatment of uterine cancer. In this report of a rare case, we describe the contribution of the appearance of mesothelial cells in the urine to the diagnosis of this condition.

**Case presentation:**

Our patient was a 71-year-old Asian woman who experienced lower abdominal pain and vomiting of two days duration. On admission, abdominal computed tomography showed intraperitoneal fluid collection and her blood tests revealed acute renal failure and hyperkalemia. She underwent hemodialysis and a transurethral catheter was inserted. The transurethral catheter was removed three days after her admission. Four days after the catheter removal, her symptoms recurred and her serum creatinine and blood urea nitrogen levels were elevated. We noted the presence of mesothelial cells in her urine, which led to a diagnosis of intraperitoneal bladder rupture. She underwent surgical repair of her bladder and hyperbaric oxygen therapy, and was discharged after her renal function returned to normal.

**Conclusion:**

Urine analysis is a simple and non-invasive test and we believe that a thorough urine analysis may contribute to the early diagnosis of an intraperitoneal bladder rupture. We think that the findings presented in this case report will significantly enhance our understanding of the etiology of bladder rupture. Moreover, these case findings may help nephrologists and urologists to rapidly diagnose this condition.

## Introduction

Spontaneous bladder rupture is an extremely rare clinical event associated with urinary ascites and apparent acute renal failure [1]. This condition is often difficult to diagnose clinically, even with increased timely access to computed tomography (CT) [2].

We report the case of a patient who presented with acute renal failure, intraperitoneal fluid collection and mesothelial cells in her urine. The presence of mesothelial cells in her urine led to the diagnosis of intraperitoneal bladder rupture.

## Case presentation

**Figure 1:**
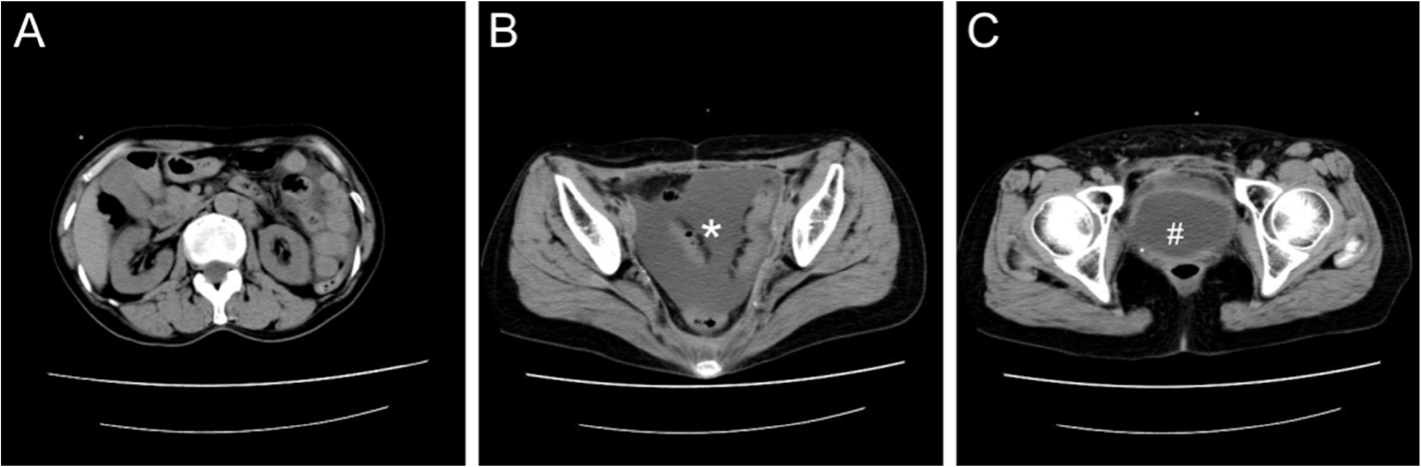
**Unenhanced computed tomography images. (A)** At the kidney level, the kidneys were normal size and hydronephrosis was not evident. **(B)** At the pelvic level, intraperitoneal fluid collection (*) was observed. **(C)** At the urinary bladder (#) level, neither blood attenuation nor bladder wall abnormalities were identified.

A 71-year-old Asian woman presented with a two-day history of lower abdominal pain and vomiting, and was admitted to our hospital. She had undergone postoperative pelvic irradiation for the treatment of uterine cancer 10 years prior to the current admission. Upon admission, her vital signs were as follows: body temperature, 37°C; heart rate, 98 beats/min; and blood pressure, 176/70mmHg. Her lung and heart sounds were normal. An abdominal examination revealed tenderness, but no muscular tension was evident; bowel sounds were audible. No edema of her extremities was observed. She presented with hyperkalemia (potassium level, 8.1mmol/L), a serum creatinine (s-Cr) level of 9.9mg/dL and a blood urea nitrogen (BUN) level of 107.2mg/dL, indicating renal failure. A work-up of her arterial blood gasses indicated metabolic acidosis (pH7.256; concentration of bicarbonate, 12.3mmol/L). Urine microscopy showed white blood cells 3 to 5 cells per high-power field (hpf) ; red blood cells, 20 to 30/hpf; hyaline casts, 8 to 10/hpf; and the presence of mesothelial cells. A urine analysis showed a value of 3+ for both protein and blood; her urine did not contain myoglobin. An abdominal CT showed intraperitoneal fluid collection, with no other abnormalities (Figure 1). After an initial evaluation, hemodialysis was initiated and a transurethral catheter was inserted.

**Figure 2:**
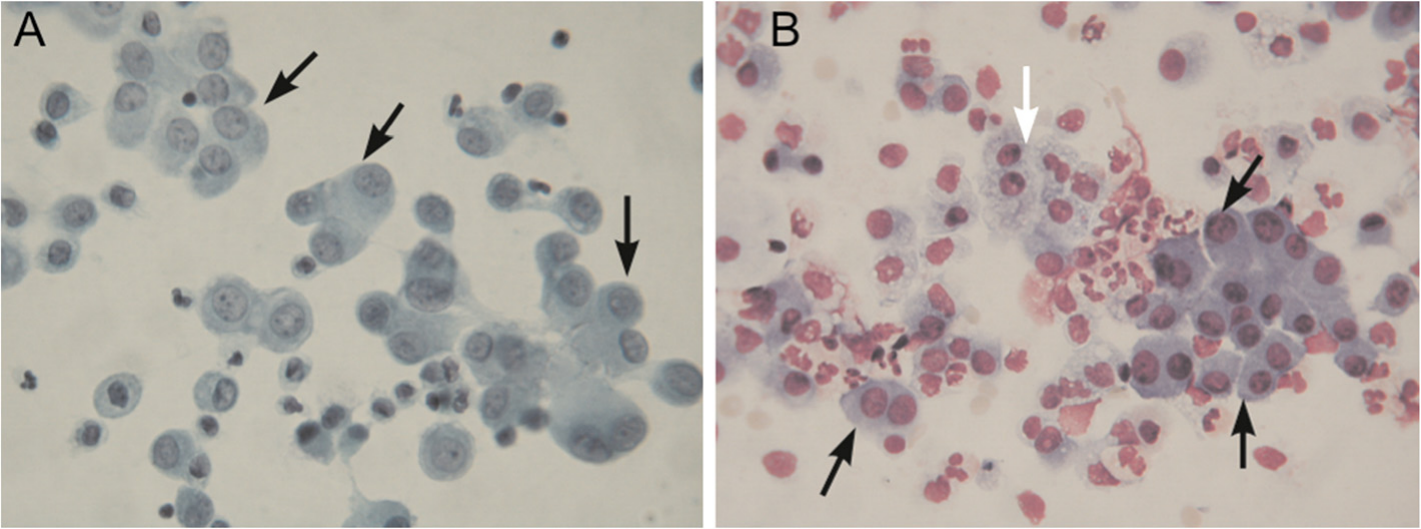
**A large number of mesothelial cells were observed in the patient’s urine. (A)** Papanicolaou staining of the patient’s urine. **(B)** Giemsa staining of the patient’s urine. Mesothelial cells are indicated by the black arrows and histiocytes by the white arrow. Magnification 400 ×.

Over the next three days, our patient’s s-Cr and BUN levels rapidly improved, and her daily urine output was good. Because her symptoms disappeared, the transurethral catheter was removed. However, four days after the removal of the transurethral catheter, her symptoms re-occurred; her s-Cr and BUN levels became elevated, and her urinary output gradually declined. At that time, we noted the presence of mesothelial cells in her urine (Figure 2), leading to the suspicion of an intraperitoneal rupture of her bladder. Cystoscopy showed a diverticulum in the apex of her bladder and a possible fistula. After the transurethral catheter was re-inserted into her bladder, her symptoms alleviated, and her s-Cr and BUN levels returned to normal.

Our patient underwent surgery to close the fistula and subsequently received 10 hyperbaric oxygen therapy sessions. The transurethral catheter was removed after these therapies and her s-Cr and BUN levels remained normal; her urine output also remained similar to that prior to the transurethral catheter removal.

## Discussion

Spontaneous urinary bladder rupture is a rare clinical event [1,3-5]. It is predominantly associated with risk factors such as radiotherapy for pelvic malignancies, neuropathic bladder, trauma, alcoholism, continuous bladder irrigation, the postpartum period and bladder diverticulum [1,4,6]. A recent report suggested that bladder rupture after radiation therapy was not as rare a complication as previously expected [7]. It might occur a long time after pelvic irradiation, as long as 30 or 40 years [1,4]. In our case, our patient had undergone pelvic irradiation 10 years before the bladder rupture occurred. Thus, her bladder rupture was likely to be caused by that treatment.

The classical symptoms of intraperitoneal bladder rupture are sudden onset of lower abdominal pain with impaired micturition [4]. Other common symptoms include nausea and vomiting, rigidity and tenderness, and difficulty or inability to void [3]. Our patient presented with some of these common symptoms, including lower abdominal pain and vomiting. In addition, patients with bladder rupture usually have urinary ascites. Autodialysis of urinary ascites across the peritoneum results in the elevation of s-Cr and BUN levels and inability to void, thus mimicking oliguric acute renal failure [1,7]. However, urinary bladder rupture associated with severe hypercreatinemia is a rare clinical presentation [7] in most cases, since transurethral catheterization promptly resolved the condition diagnosed on the basis of laboratory findings of oliguric acute renal failure [5]. To date, only a few other patients have been reported to require hemodialysis because of acute renal failure caused by bladder rupture [7,8]. In our case, because our patient’s abdominal pain was initially not very severe, she did not visit our hospital until two days after the pain began. Therefore, on admission she presented with severe, life-threatening hyperkalemia and uremia that required hemodialysis.

For spontaneous bladder rupture, the mortality rate associated with a delay in diagnosis of 24 hours or more is as high as 25%. However, this mortality rate can be minimized by appropriate preoperative diagnosis and prompt surgical correction [4]. A finding of intraperitoneal fluid collection in CT may be useful to detect bladder perforation at an earlier stage but this finding alone does not warrant a definitive diagnosis [9]. Although CT cystography might also be beneficial for the detection of bladder perforation [3], it is difficult to perform in a patient with acute renal failure. Measuring urea and creatinine levels in urine ascites and plasma is a simple and non-invasive diagnostic test to diagnose spontaneous bladder rupture [10]. We did not perform a peritoneal fluid analysis because we did not initially consider the possibility of a spontaneous bladder rupture. Our patient’s s-Cr and BUN levels rapidly improved and her symptoms disappeared after hemodialysis and transurethral catheter implantation, but her urine volume gradually decreased and her symptoms redeveloped after the removal of the transurethral catheter. At that time, we noted the presence of a large number of mesothelial cells in her urine; this finding was suggestive of an intraperitoneal rupture of the bladder. Kumagai *et al*. previously reported the occurrence of mesothelial cells in the urine of a patient with spontaneous bladder rupture, as in our case [11]. Thus, a thorough urine analysis may contribute to the early diagnosis of intraperitoneal bladder rupture.

## Conclusion

In cases where mesothelial cells are observed in the patient’s urine, the possibility of an intraperitoneal bladder rupture should be considered. A urine analysis is a simple and non-invasive test that may contribute to the early diagnosis of this condition.

## Consent

Written informed consent was obtained from the patient for publication of this case report and the accompanying images. A copy of the written consent is available for review by the Editor-in-Chief of this journal.

## Abbreviations

BUN: blood urea nitrogen; CT: computed tomography; hpf: high-power field; s-Cr: serum creatinine.

## Competing interests

The authors declare that they have no competing interests.

## Authors’ contributions

WH, SN and TN were the major contributors in writing the manuscript. WH and SN prepared the figures. TN performed the literature review and performed the final editing of the manuscript. WH and SN performed the clinical examination of the patient. WH, SN and YO interpreted and diagnosed the cytological findings. MF performed the surgical procedure. YO and SK reviewed and discussed the manuscript. All authors read and approved the final manuscript.
